# Does Percutaneous Kyphoplasty Have Better Functional Outcome Than Vertebroplasty in Single Level Osteoporotic Compression Fractures? A Comparative Prospective Study

**DOI:** 10.1155/2013/690329

**Published:** 2013-07-18

**Authors:** F. Omidi-Kashani, F. Samini, E. G. Hasankhani, A. R. Kachooei, K. Z. Toosi, F. Golhasani-Keshtan

**Affiliations:** ^1^Orthopedic Research Center, Orthopedic Department, Imam Reza Hospital, Faculty of Medicine, Mashhad University of Medical Sciences, Mashhad 9137814864, Iran; ^2^Department of Neurosurgery, Emdadi Hospital, Faculty of Medicine, Mashhad University of Medical Sciences, Mashhad, Iran; ^3^Orthopedic Department, Ghaem Hospital, Faculty of Medicine, Mashhad University of Medical Sciences, Mashhad, Iran; ^4^Department of English, University of Birjand, Birjand, Iran; ^5^Orthopedic Research Center, Mashhad University of Medical Sciences, Mashhad, Iran

## Abstract

*Purpose*. To evaluate the relative differences in surgical outcome of kyphoplasty (KP) versus vertebroplasty (VP) in the patients with single level refractory osteoporotic compression fractures (OCFs). *Method*. From August 2008 to May 2012, we intermittently treated 57 patients with single level OCF by PV and KP (Groups A and B, resp.). We used visual analogue scale (VAS) and short form 36 (SF36) questionnaire to measure functional recovery and followed them for six months. Independent samples *t*- and Kendall's tau-b tests were for statistics. *Results*. In terms of age, number, and bone mineral density of the patients, there were no significant differences between the two groups. In both groups, VAS and SF-36 scores improved significantly and remained relatively stable throughout the follow-up period. We had 9 and 6 asymptomatic cement extravasations and 5 and 8 new vertebral fractures in Group A and B, respectively. In comparing the two groups, the results indicated that KP almost failed to show any significant higher effect relative to VP during this period. *Conclusions*. In considering the high cost of KP relative to VP in the developing countries like Iran, there is no logical reason to use KP in a single level refractory OCF in these regions.

## 1. Introduction

Osteoporotic compression fractures (OCFs) are common debilitating entities that cause a variety of consequences in the elderly patients. Although two-third of them gradually improve with conservative treatment [1], intractable pain, decreased self-esteem, senile kyphosis, early satiety, mood disorder, and even increased mortality have been frequently reported [2–5]. 

Today vertebral augmentation procedures (vertebroplasty (VP) and kyphoplasty (KP)) are commonly used in the treatment of otherwise unmanageable OCFs [6–8]. Potency and effectiveness of these procedures have repetitively been proven in numerous articles [1, 9–12]. Due to higher viscosity of the cement during KP procedure, it theoretically seems that its cement leakage incidence would be less than VP [13, 14]. Moreover, restoring vertebral body height and alignment may make KP more attractive among the spinal surgeons, while this method is more time consuming and expensive [7, 8, 10, 13, 15]. Can a single level vertebral height restoration really be associated with better patient's quality of life? The aim of this prospective study was to evaluate the relative differences that are present in surgical outcome (quality of life and pain intensity) of KP versus VP in the patients with single level refractory OCFs. 

## 2. Materials and Methods

From August 2008 to May 2012, we prospectively treated and followed 64 patients with single level OCF by percutaneous cement augmentation. After obtaining institutional review board approval, we intermittently treated the patients with VP (Group A) and KP (Group B). Our inclusion criteria for this project were single level osteoporotic vertebral fracture [16] with more than 3 to 4 weeks old of age [9], localized pain refractory to medical treatment, concordance of local pain and injured vertebra in clinical examination [14], and magnetic resonance [17] or bone scan imaging [18] findings that have confirmed the acuity of the fracture.

We exclude the asymptomatic patients, those who had an appropriate response to medical treatment, the patients with significant and especially progressive neurologic deficit, those with underlying diseases that might have affected the process (like allergy to bone cement, unmanageable hemorrhagic disorders, infection, or tumor), the patients with vertebral collapse more than two-third of the primary vertebral height, and those with less than 6 months follow-up period. 

Bone mineral density was preoperatively measured as an indicator of osteoporosis with dual-energy X-ray absorptiometry in all the cases. We only included the osteoporotic patients (according to the World Health Organization; these patients have a T-score −2.5 or lower, meaning a bone density that is two and a half standard deviations below the mean of a thirty-year-old man/woman) [16]. 

Two spinal scaling questionnaires in our patients were used to measure functional recovery. Overall pain was measured on a numerical vertical scaling line (visual analogue scale (VAS)) from 0 (no pain at the base) to 10 (maximal imaginable pain at the summit) [19]. We assessed the patients' health status with the Short Form 36 (SF36) questionnaire [20]. Translation and validation study of the Iranian version of the form has already been performed by Montazeri and coauthors [21]. The form evaluates physical and mental health status covering eight subscales: physical function (PF), social functioning (SF), role physical (RP), role emotional (RE), mental health (MH), vitality (VT), bodily pain (BP), and general health (GH). Finally, the scores for the eight subscales were calculated according to the algorithm described in the SF-36 manuals [22]. The questionnaires were completed preoperatively, 4 weeks, and 6 months after surgery. We also recorded various intra- and postoperative radiologic and clinical complications encountered with these procedures. 

### 2.1. Surgical Technique

All cement augmentation procedures were performed in the operating room with availability of probable immediate decompressive surgery. The patient was positioned prone on two transverse bolsters under chest and pelvis. Then, we applied a gentle three-point reduction force on the fractured vertebra to relatively reduce the kyphotic posture. General anesthesia and bipedicular access were routinely used in KP group, but in VP patients, we sometimes used only sedation under anesthesiologist supervision and unilateral approach. In the latter group, we aimed to get a relatively widespread distribution of cement, and this goal might be achieved by unilateral or bilateral transpedicular approach. We used polymethylmethacrylate (PMMA) cement with Stryker Precision Cement Delivery System and KyphX Inflatable Bone Tamps (Kyphon, Inc., Sunnyvale, CAUSA) in VP and KP patients, respectively. Other, points of surgical technique were similar to what has been mentioned previously [10, 23–25].

### 2.2. Statistical Analysis

Calculations were performed using the software package for statistical analysis (SPSS) version 11. The independent samples *t-*test was used to compare the mean scores of the two groups on a given variable. A *P* value of less than 0.05 was considered significant in all our data analysis. We also used Kendall's tau-b to measure the association between complication and type of the procedure. 

## 3. Results

**Figure 1:**
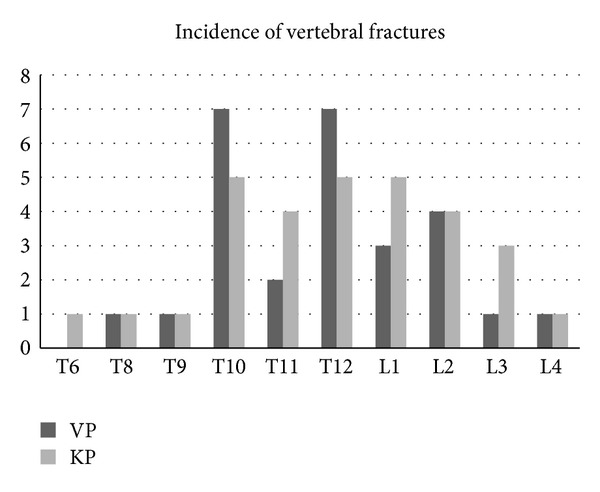
Incidence of vertebral fractures in the patients (both groups).

Seven patients were excluded due to inadequate followup, and finally we studied 57 cases. In Group A (VP patients), there were 28 patients (male to female: 6 to 22) with a mean age of 72.4 ± 8.2 years (range, 60.1 to 91.3 years), while in Group B (KP patients) these parameters were 29, 7 to 22, 72.1 ± 6.2, and 62.2 to 86.3 years, respectively. In terms of age and number of the patients, there were no significant differences between the two groups (*P* = 0.8 and *P* = 1, resp.). Incidence of vertebral fractures in our patients is shown in Figure 1.

The average bone mineral density T score for the lumbar spine in Groups A and B were −2.9 and −2.7, respectively, with no significant difference between the two statistically. The mean volume of the injected cement in Group A (VP) and Group B (KP) was 3.5 ± 0.4 mL (ranged from 2.2 to 4.3) and 5.1 ± 0.9 mL (ranged from 3.2 to 7.1), respectively (*P* < 0.001). The difference between the two was statistically significant (*P* < 0.001). In Group A, the mean VAS score decreased from 7.6 ± 1.2 preoperatively to 1.7 ± 0.1 four weeks (*P* < 0.001) and 1.6 ± 0.8 in six months after VP. Pain remained low between the first and six months after VP (*P* = 0.75). In Group B, these scores were 7.2 ± 1.4, 1.8 ± 0.9 (*P* < 0.001), and 1.8 ± 1.2 (*P* = 1.4), respectively. Statistical comparison of VP with KP in the term of pain relief in these patients suggested that the difference in the first and sixth month after surgery was not significant (*P* = 0.58 for the first and 0.50 for the sixth month). 

Quality of life measured with SF-36 questionnaire improved substantially and remained high in both groups throughout the six months follow-up period (Table 1). Both VAS and SF-36 scores demonstrated statistic similarity between the two groups, preoperatively. In comparing the two groups in term of quality of life, the results suggested that KP almost failed to show any significant different effect relative to vertebroplasty (except in physical functioning) in four weeks and six months postoperatively (*P* = 0.24 and 0.07, resp.). In four weeks and six months after operation, physical functioning domain was significantly higher in VP cases. 

We arbitrarily divided our surgical complications into intraoperative and postoperative ones. These adverse events are also shown in Table 2. None of our cement extravasation evens were associated with clinical symptoms. As the table shows, most of the recently fractured vertebrae (80% in VP and 75% in KP patients) had occurred adjacent to the formerly cemented ones. Based on Kendall's tau-b test, with a correlation coefficient of −0.007 and *P* value 0.964, no significant correlation exists between the incidences of complications of surgery with the type of surgery (VP versus KP).

## 4. Discussion

In this research, we were able to study and follow two relatively homogeneous groups of patients with single level refractory OCF treated with VP and KP. Our findings confirmed the results of previous studies, showing that these two procedures are relatively safe and well able to reduce pain and improve patients' quality of life, although we could not find a significant advantage of KP compared to VP during the six months of followup. 

In a similar research, De Negri et al. conducted vertebroplasty and kyphoplasty in a prospective nonrandomized controlled study on 21 patients with painful compression fracture and followed them for six months [15]. They included both osteoporotic and traumatic vertebral fractures and evaluated them with VAS and Oswestry Disability Index (ODI) [26, 27]. The average pre- to postoperative VAS scores for VP and KP patients were 8.4 to 0.6 and 8.3 to 0.7, respectively. Similarly, the average pre- to postoperative ODI for VP and KP groups was 74% to 24% and 77% to 23%. Based on their results, they suggested that both of the procedures have the same significant therapeutic effect on decreasing pain and recovering function. They reported that cement extravasation has occurred only in vertebroplasty group, but in the study we conducted, we observed 4 cases (13.8%) with leakage of cement in kyphoplasty patients. 

Han et al. in a systematic review and meta-analysis study compared the efficacy and safety of VP with KP for treatment of OCFs [8]. They only included those papers that had used VAS and ODI to evaluate the clinical outcomes regardless of the number of vertebrae involved. In long-term pain relief, functional improvement, and complication (cement leakage and further fracture) rate, they also observed no significant difference between the two procedures. Due to higher price of KP compared to VP, the researchers offered the latter for the treatment of OCFs. As we noted, in this study, the researchers did not consider the effect of the number of involved vertebrae on the clinical outcome. Unlike our study, in this research, no attention was paid to the number of the fractured vertebrae and its probable confounding role in postoperative clinical improvement. 

In a similar way, Ma and coauthors also compared these two procedures to treat OCFs in 1081 patients through a systematic review and meta-analysis [7]. They found that although both of these methods are safe and effective, in a subgroup of patients with severe kyphosis, posterior vertebral body fracture or fissure, and associated significant vertebral body collapse, KP may be preferred. These authors ultimately emphasized the necessity of high-quality randomized controlled trial studies to reduce uncertainty in this issue. In a cost-effectiveness study in the patients hospitalized with acute OCFs, three methods of VP, KP, and conservative management were compared and cost-effectiveness, mortality, and health-related quality of life were assessed [6]. Although this study was conducted in UK and may not be applicable to other countries, the authors suggested KP as the most cost-effective method to treat these especial hospitalized patients.

Another persisting concern is an increased risk of new fracture in adjacent vertebrae. This can be attributed to biomechanical changes of the augmented vertebra relative to others [28]. According to our knowledge, no author has been able to conclusively prove that cement augmentation compared to nonsurgical treatment is associated with an increased risk of new vertebral fractures in the future. Many of these osteoporotic patients, even without any vertebral augmentation procedures, will fracture more in the coming months or years [12, 29, 30]. In our patients, 5 and 8 new vertebral fractures occurred in 28 and 29 patients in Groups A and B, respectively, that there was no significant difference between the two groups. 80% of the newly fractured vertebrae in VP and 75% in KP patients had occurred adjacent to the previously augmented vertebrae. In this field, Uppin et al. have carried out a research in 177 cases with OCFs [29]. All of these cases were treated with vertebroplasty and followed for more than two years. The researchers observed 36 new vertebral fractures in 22 patients, while 67% of these new fractures have occurred within 30 days after VP and adjacent to the previously augmented segment. In another study, Grados and colleagues evaluated long-term (mean follow-up period 48 months) outcome of PV in the patients with OCFs [12]. During this period, 18% of cases had new vertebral fractures. The odds ratio of an adjacent compared with nonadjacent vertebral fracture was 2.27 and 1.44, respectively. Therefore, it is generally recommended that after PV, the patients should be frequently seen to look for new vertebral fractures and the necessity of treatment change, accordingly [11]. These studies were related to the VP patients, but Campbell and Harrop in a review of the literature evaluated incidence of adjacent level fractures in the patients treated with KP [30]. They ultimately failed to find a strong link between KP and its impact on the incidence of adjacent vertebral fractures as well. 

This research has some limitations. Although the study was carried out prospectively, the number of the cases was relatively small, and the efficacy of the associated conditions like probable pre- and postoperative antiosteoporotic drugs was not perfectly controlled. Therefore, a logical deduction might not be strongly recommended. In conclusion, although theoretically KP with restoring vertebral height and improving sagittal alignment should have better clinical and radiological effect in patients with OCFs, its clinical preference has not been established. In considering the high cost of KP relative to VP in developing countries, it might be better to use VP in single level refractory OCF in these territories.

## Figures and Tables

**Table 1:** Comparison of the effects of VP and KP on eight domains of SF-36 in our patients.

Domain	Mean score (SD^#^) in VP versus KP group	*P* value
Physical functioning (PF):		
Preop	32.3 (7.8)–35.5 (8.1)	0.13
4 w^$^ postop	73.9 (10.5)–64.1 (8)	0.00*
6 m^§^ postop	73 (11.6)–62.9 (11.6)	0.002*
Role physical (RP):		
Preop	21.2 (17.6)–24.1 (19.4)	0.56
4 w postop	75 (15.2)–77 (13.9)	0.50
6 m postop	75.8 (10.7)–75 (13.3)	0.89
Bodily pain (BP):		
Preop	30.7 (10.2)–32 (11.6)	0.66
4 w postop	71.8 (10.8)–70.2 (10.5)	0.56
6 m postop	74.8 (10.3)–72.4 (13.2)	0.45
General health (GH):		
Preop	59.5 (9.0)–60.8 (9.6)	0.60
4 w postop	75.6 (5.7)–74.0 (6.5)	0.31
6 m postop	74.2 (7.9)–73.5 (6.8)	0.71
Vitality (VT):		
Preop	51.6 (12.4)–51.8 (12.5)	0.93
4 w postop	66.2 (10.8)–68.7 (12.7)	0.41
6 m postop	69.1 (9.7)–67.9 (15.7)	0.73
Social functioning (SF):		
Preop	58.4 (14)–58.6 (15.3)	0.97
4 w postop	75.8 (12.2)–73.7 (14.3)	0.13
6 m postop	76.3 (14.5)–70.2 (15.8)	0.13
Role emotional (RE):		
Preop	51.4 (28.9)–59.7 (28.7)	0.28
4 w postop	69.3 (24.9)–72.4 (21.9)	0.61
6 m postop	70.2 (20.9)–68.9 (25)	0.83
Mental health (MH):		
Preop	63.5 (10.9)–66 (11.7)	0.41
4 w postop	73 (11.1)–77.7 (8.5)	0.07
6 m postop	72 (10.4)–72.5 (13.2)	0.97
Total score SF-36:		
Preop	45.8 (7.7)–47.3 (5.5)	0.39
4 w postop	73.1 (5.2)–71.5 (5.01)	0.24
6 m postop	73.02 (7.0)–69.3 (8.2)	0.07

^#^SD: standard deviation.

^$^w: week.

^§^m: month.

*Statistically significant.

**Table 2:** Complications of cement augmentation procedures in our patients.

Type of complication	Incidence in VP (%)	Incidence in KP (%)
Intraoperative:		
Cement extravasation into disc space	6 (21.4)	2 (6.9)
Cement extravasation into paravertebral space	1 (3.6)	2 (6.9)
Cement extravasation inside injection tract	3 (10.7)	0 (0)
Rib fracture	1 (3.6)	2 (6.9)
Postoperative:		
Superior adjacent vertebral fracture	1 (3.6)	2 (6.9)
Inferior adjacent vertebral fracture	3 (10.7)	4 (13.8)
Nonadjacent vertebral fracture	1 (3.6)	2 (6.9)
